# Workplace Demands, Control, and Identification as Predictors of Job Satisfaction

**DOI:** 10.3390/ejihpe16010009

**Published:** 2026-01-05

**Authors:** Samuel Fernández-Salinero, Giulia Foti, Gabriele Giorgi, Gabriela Topa, Pablo Garmendia

**Affiliations:** 1 Department of Psychology, Rey Juan Carlos University, 28922 Madrid, Spain; 2 Department of Human Sciences, European University of Rome, 00163 Rome, Italy; giulia.foti@unier.it (G.F.); gabriele.giorgi@unier.it (G.G.); 3 Department of Social and Organizational Psychology, Universidad Nacional de Educación a Distancia (UNED), Juan del Rosal, 10, 28040 Madrid, Spain; gtopa@psi.uned.es; 4 International School of Doctorate (EIDUNED), National Distance Education University (UNED), 28040 Madrid, Spain; pgarmendi4@alumno.uned.es

**Keywords:** job satisfaction, job demands, job resources, organizational identification, workgroup identification

## Abstract

Job satisfaction is a key component of organizational well-being and performance. This study explores the relationship between job characteristics (demands and resources), organizational and group identification, and affective job satisfaction, drawing on the Job Demands–Resources model and social identity theory. Methods: A cross-sectional design was used with a sample of 420 employed individuals (49% men and 51% women), with a mean age of 41.58 years (SD = 9.95). Participants completed an online questionnaire assessing job demands (quantity and complexity), job resources (competency development, use of competencies, and decision latitude), and identification (organizational and group). Most participants held a university degree (48.1%) and had a permanent contract (79.8%). A MIMIC model was applied to test the hypothesized relationships. Results: Job demands defined by quantity were inversely associated with job satisfaction, while competency development and use were positively associated. Organizational and group identification also showed significant positive relationships with job satisfaction. However, task complexity and decision latitude were not significant predictors. Discussion: These findings underscore the importance of integrating both job design and social identity factors to enhance job satisfaction and inform organizational strategies aimed at promoting employee well-being.

## 1. Introduction

Job satisfaction is one of the variables that has most enriched scientific literature on organizational psychology. It has been examined from multiple perspectives, and numerous management strategies have been proposed to promote it (Judge et al., 2002; Spector, 1997). Its relevance stems from the consistent associations found with positive organizational outcomes such as performance, commitment, and well-being (Wright et al., 2002; Kuvaas, 2006). These relationships make job satisfaction one of the key components of competitive, viable, and healthy organizations (Schneider et al., 2013).

Job satisfaction is defined as a positive emotional state stemming from an employee’s evaluation of their work or work experiences (Locke, 1976). This variable has been highly prolific in organizational literature and remains central in contemporary studies on workplace well-being (Quesada-Puga et al., 2024). Previous research has demonstrated that job satisfaction is a broad phenomenon, encompassing both cognitive and affective dimensions (Fernández-Muñoz & Topa, 2018). Given that organizations are complex structures, it is not possible to establish linear relationships between a single factor and job satisfaction. Instead, integrated models, such as the Job Demands–Resources (JD-R) model, have been employed to explain how multiple factors—such as tasks, salary, growth opportunities, and social environment—interact to influence satisfaction (A. B. Bakker & Demerouti, 2017; Schaufeli, 2021). These factors do not operate independently but interact with one another, creating complex dynamics that make it more challenging to predict and fully understand the phenomenon of job satisfaction (Van De Voorde et al., 2016).

Recent studies have highlighted the role of job satisfaction as a significant predictor of work performance (Katebi et al., 2022; Inayat & Jahanzeb Khan, 2021). Furthermore, recent findings emphasize that work environments promoting job satisfaction are linked to decreases in undesirable variables such as absenteeism (Ybema et al., 2010) and turnover intentions (Alam & Asim, 2019).

Since the origins of the scientific organization of work, enhancing organizational performance and scientifically understanding how various organizational characteristics influence this variable has been a recurring objective. One of the most extensively studied variables in this context has been the nature of the job itself. In this vein, Job Demands-Control (JD-C) model (R. A. Karasek, 1979) provides a valuable lens for understand how job characteristics can affect job satisfaction. The JDC model aimed to predict how the level of job demand, in combination with the degree of control employees have over their work affect their job satisfaction. However, scientific literature demonstrated that job satisfaction is a more complex phenomenon that cannot be fully explained by simplistic factors or strictly linear models (Van De Voorde et al., 2016; A. B. Bakker & Demerouti, 2017; Judge et al., 2002).

For this reason, analyzing solely the demands of the job and the corresponding control to face them has proven insufficient in explaining job satisfaction. Other job resources, such as organizational and group identification, also contribute to shape employees job satisfaction. Furthermore, employees’ identification with their organization is positively related to commitment and job satisfaction, which enhances performance and reduces negative behaviors such as turnover and absenteeism. Moreover, greater organizational identification leads to higher employee engagement, particularly in contexts where leadership fosters an empowering environment (Arshad et al., 2022). Similarly, it has been demonstrated that identification with the workgroup also significantly impacts satisfaction. Previous studies have shown that high-performance practices in organizations can positively affect variables such as engagement, with this relationship being mediated by organizational identification (Salin et al., 2023).

Van De Voorde et al. (2016) emphasize that comprehending job satisfaction requires a multi-level approach, considering not only individual aspects but also the social and organizational dynamics that interact with one another. In the current context, where the labor market is undergoing significant changes due to globalization and hyperconnectivity, it is crucial to understand what contributes to greater employee satisfaction. Studies such as those by Ellemers et al. (2014) and Riketta and Van Dick (2005) have demonstrated that organizational and group identification are key factors affecting not only employee performance and well-being but also commitment, productivity, motivation, and turnover, among others. These elements, combined with personal resources (such as job control), provide a more comprehensive understanding of job satisfaction. This approach brings us closer to a holistic and complete view of the phenomenon, which can help managers and leaders develop a more realistic and accurate understanding of organizational reality.

Having said this, the main objective of this research is to explore the relationship between job characteristics (job demands and job resources), organizational and workgroup identification, and job satisfaction. Specifically, we will examine the relationship between job demands, control, organizational and group identification, and affective job satisfaction.

## 2. Theoretical Framework

### 2.1. Job Content: Karasek’s Job Demand-Control Model

Analyzing work is a complex task that has been approached from various perspectives. In an initial attempt to describe job content, R. Karasek et al. (1998) proposed that work is defined by a series of psychological and physical demands that individuals must address using specific resources. To carry out these tasks, workers require various resources, including material, social, and skill-based resources. It is essential to note that, to complete a job, individuals must be able to develop their skills and have the opportunity to use them.

R. A. Karasek (1979) developed a model that combined the demands placed on workers with the decision-making latitude they had to address them. For Karasek, citing Quinn et al. (1971), differences in job satisfaction were not due to the demands themselves but to the individual’s ability to make decisions.

Job demands are aspects of work that require sustained effort and are associated with physiological and psychological costs. These demands can vary in complexity, intensity, or quantity. The quantity of job demands refers to the workload an employee faces, while complexity and intensity relate to the difficulty of the tasks they must perform. This is reflected in the analyses of Kahn (1973), who distinguishes between quantitative and qualitative demands. R. A. Karasek (1979), on the other hand, argued that while all demands may appear inherently stressful, the reality is more nuanced.

In contrast, job resources are aspects that have motivational potential, help achieve work goals, regulate the impact of demands, and stimulate personal growth. Resources can be material, temporal, social, or personal. Initially, R. A. Karasek (1979) referred to resources as decision-making latitude (such as autonomy in task execution, etc.), proposing that this factor relates to the ability to transform the stress caused by demands into action. At this point, the author describes occupational stress as arising from jobs with high demands and low decision-making latitude. It is here that we can observe an interaction between personal and organizational factors. Naturally, as the model shifted from an individual focus on stress, the analysis of job components became an important consideration in subsequent work.

### 2.2. Job Content: Bakker and Demerouti’s Job Demands–Resources Model

The Job Demands–Resources (JD-R) model proposed by A. Bakker and Demerouti (2013) builds upon the previously mentioned Job Demand-Control model. Both models share the idea that job content significantly impacts employee well-being and performance. However, the JD-R model introduces additional contributions that complement and expand Karasek’s perspective. The authors define job resources as all aspects of work that can help employees achieve their goals (A. B. Bakker & Demerouti, 2017).

The JD-R model broadens the definition of job resources, distinguishing between job-related resources and personal resources (A. B. Bakker & Demerouti, 2017). Specifically, job resources (e.g., competency use or decision latitude) foster satisfaction by triggering motivational processes that can help employees find meaning in their work. The authors propose that job demands and resources do not operate independently but interact, creating a dynamic balance between job demands and resources, both from the workplace and the individual. For example, in terms of personal resources, competencies refer to the skills, knowledge, and attitudes employees need to perform their jobs effectively. These competencies may be developed prior to employment or within the organization. In any case, these competencies help mitigate the stressful impact of demands. However, possessing a specific competency does not guarantee its utilization. Job design must align the level of demands with the employee’s personal characteristics and the organization’s resources. If there is a mismatch, work overload could lead to burnout (A. B. Bakker & Demerouti, 2017). The work environment must provide employees with opportunities to develop resources and apply their skills.

Recent scientific literature confirms that workload and task intensity significantly impact employee job satisfaction (Hellín Gil et al., 2022). Excessive workload and unreasonable tasks can generate stress, emotional exhaustion, and a diminished sense of control, thereby reducing job satisfaction. Recent studies have shown that intense work rhythms increase mental fatigue and emotional exhaustion, leading to lower satisfaction and well-being among employees and, in some cases, fostering psychological detachment from work (Mihelič et al., 2024; Gottwald & Lejsková, 2023; Schaufeli & Bakker, 2004).

On the other hand, when tasks are well-distributed and sufficiently challenging without being overwhelming, they generate greater motivation and satisfaction. The JD-R model suggests that an appropriate balance between workload and available resources (such as support or autonomy) can prevent burnout and foster a more satisfying work environment (A. B. Bakker & Demerouti, 2007; Hakanen et al., 2006). Additionally, recent reviews have proposed that when employees face stressful tasks, they may resort to maladaptive coping strategies that lead to emotional exhaustion (A. B. Bakker & De Vries, 2021).

The analysis of job resources can be approached from several perspectives. A. B. Bakker and Demerouti (2017) emphasize that resources exist at different levels. First, there are personal resources, such as the ability to influence one’s work or control. For example, the ability to develop competencies and the opportunity to use them are two factors included in this study that operate at the individual level. Second, team-level resources must also be considered. In our research, we have selected group identification as a team-level resource that can help employees cope with demands. Finally, organizational resources refer to aspects such as human resource practices, culture, or performance. In our study, we consider that organizational identity can also act as a significant resource in addressing demands.

Therefore, the current research aims to understand the differential role of various types of demands and resources, analyzing the social component through organizational identification.

### 2.3. Development and Use of Competencies and Job Satisfaction

As mentioned in the previous section, personal resources play a role similar to job resources in addressing demands. Personal resources are defined as the beliefs employees hold about the extent to which they have control over their work (A. B. Bakker & Demerouti, 2018). The ability and opportunity to develop competencies at work can be understood as a valuable personal resource for tackling job tasks. Previous research has shown that when employees have the opportunity to use their skills, they exhibit higher levels of job satisfaction (Morrison et al., 2005; Mateos Romero & Salinas-Jiménez, 2018).

On the other hand, a high level of job demands and a low level of resources (both job-related and personal) are associated with a greater risk of burnout. Again, we can observe the dynamic balance that must exist between demands and resources. If demands exceed the individual’s resources, and this situation persists over time, symptoms of burnout may arise. Burnout is characterized by emotional exhaustion, depersonalization, and work-related cynicism, and it can have serious consequences for employees’ physical and mental health (Maslach & Jackson, 2013; Leiter & Maslach, 2011).

When employees have the opportunity to develop and apply their competencies in a work environment where the balance between demands and resources is appropriately managed, the risk of burnout is significantly reduced. Recent studies on the Job Demands–Resources (JD-R) model show that access to job resources such as support and autonomy, along with the opportunity to use acquired competencies, increases the sense of control and mastery over tasks. This helps mitigate the negative effects of stress and improves well-being and job satisfaction (Xanthopoulou et al., 2007).

Conversely, the inability to use developed competencies, especially when job demands are high and resources are scarce, can increase the perception of lack of control, raising the likelihood of emotional exhaustion and reducing job satisfaction. In such cases, employees often experience more stress and frustration due to the dissonance between their capabilities and the actual opportunities to apply them, which can lead to greater burnout symptoms and lower job satisfaction (Kristof-Brown et al., 2005).

### 2.4. Organizational Identification

In the field of organizational psychology, two closely related but distinct concepts have gained prominence in recent years: organizational identification and workgroup identification. Although both concepts refer to the extent to which an individual identifies with a social group within the organization, there are important differences that must be understood to analyze their impact on individual and organizational behavior.

First, organizational identification is defined as the extent to which an individual identifies with an organization and considers themselves part of it. This type of identification is characterized by a sense of belonging to the organization, pride in being part of it, and commitment to its goals. Current studies have shown that organizational identity is a crucial variable in the survival of organizations, favoring positive outcomes (Newman et al., 2016).

On the other hand, workgroup identification is defined as the extent to which an individual identifies with their immediate workgroup and considers themselves part of it (Hogg & Turner, 1985). Workgroup identification has been shown to significantly impact team cohesion and group performance (Riketta & Van Dick, 2005). In previous research, we have demonstrated that workgroup identification is significantly related to job satisfaction (Fernández-Salinero et al., 2020). However, we have also analyzed in prior studies that identities can conflict, having detrimental effects on organizational outcomes (Fernández-Salinero et al., 2020; Fernández-Salinero & Topa, 2020).

The main difference between organizational identification and workgroup identification lies in the scope of identification. Organizational identification refers to identification with the organization as a whole, while workgroup identification refers to identification with a specific group within the organization, such as a team or department (Hogg & Terry, 2000). The dynamics of both types of identification can also change over time depending on various factors, such as the individual’s experience in the organization, the performance of the workgroup, and organizational culture (Haslam et al., 2004). These identities can be aligned or in conflict, depending on their current state.

Both organizational identification and workgroup identification have significant consequences for individual well-being, job performance, and organizational success. In terms of individual well-being, employees with higher levels of identification tend to experience greater psychological well-being, lower stress, and higher job satisfaction (Ellemers et al., 2014). Additionally, they feel more valued and supported, which contributes to better mental and physical health (Mael & Ashforth, 1992). However, it is necessary to investigate both types of identification, as they operate at different levels of inclusion and represent distinct sources of job resources that, as mentioned earlier, can either reinforce or hinder each other.

Finally, in relation to job performance, employees who identify with the organization and their workgroup tend to be more productive, creative, and committed to their work and the organization, and they exhibit lower absenteeism and turnover (Van Dick et al., 2004). Furthermore, they are more willing to collaborate with their colleagues and strive to achieve both group and organizational goals (Korte, 2007).

### 2.5. Job Satisfaction

Job satisfaction reflects the subjective evaluation that employees make about various aspects of their job, such as the tasks they perform, working conditions, relationships with colleagues and supervisors, development opportunities, and the recognition they receive (Spector, 1997).

Job satisfaction is a multidimensional construct comprising different components. One of the most studied components is satisfaction with the work itself, which refers to the degree of satisfaction employees experience with the tasks they perform, the level of challenge these tasks present, and the meaning they find in their work (Hackman, 1976). Another crucial component is satisfaction with working conditions, which includes aspects such as salary, benefits, work environment, job security, and work–life balance (Warr, 1987).

Additionally, satisfaction with interpersonal relationships refers to the degree of satisfaction employees experience with the relationships they maintain with their coworkers, supervisors, and clients (Cropanzano et al., 2017). Satisfaction with development opportunities is another important component, related to the degree of satisfaction employees experience with the opportunities they have to learn new skills, grow professionally, and take on new responsibilities (Super & Hall, 1978). Finally, satisfaction with recognition refers to the degree of satisfaction employees experience with the recognition they receive for their work, both from their peers and supervisors (Deci & Ryan, 2000).

Recent research has shown that employees with high job satisfaction tend to experience greater psychological well-being, lower stress, better physical health, and an overall higher quality of life (Judge et al., 2002). A meta-analysis by Çivilidağ et al. (2024) found significant negative relationships between job satisfaction and both work stress and burnout among healthcare workers during the COVID-19 pandemic, highlighting the protective role of job satisfaction against psychological distress. Furthermore, job satisfaction influences job performance. Satisfied employees tend to be more productive, creative, committed to their work and the organization, and exhibit lower absenteeism and turnover (Wright et al., 2002; Mascarenhas et al., 2022). Higher job satisfaction among employees can contribute to a better organizational image, greater customer loyalty, and increased financial success (Kuvaas, 2006; Hosseini & Ferreira, 2023).

Despite the extensive literature on job satisfaction, many studies tend to analyze its predictors in isolation, focusing either on job characteristics or on social identification, but rarely integrating both perspectives into a comprehensive model. Additionally, although organizational and group identification have been associated with positive outcomes, few studies have examined their simultaneous contribution to affective job satisfaction using an integrative statistical model. We have chosen to investigate individual, structural and psychosocial factors to gain a deeper perspective of this phenomenon. This study aims to address these gaps by jointly analyzing job content variables and social identity factors, using a MIMIC model to provide a multidimensional understanding of how they relate to affective job satisfaction. In doing so, we contribute to a more nuanced understanding of the interplay between structural and psychosocial elements in shaping employees’ well-being.

Numerous factors can influence job satisfaction, both individual and organizational. Among the most important individual factors are personal characteristics, such as personality, values, expectations, and motivations, which can influence an employee’s level of job satisfaction (Judge et al., 2002). On the other hand, employees who feel competent and capable of performing their work effectively tend to be more satisfied (Hackman, 1976). Additionally, job satisfaction depends on the extent to which the job meets the employee’s needs and desires, both in terms of intrinsic aspects, such as challenge and meaning, and extrinsic aspects, such as salary and benefits (Deci & Ryan, 2000).

Among the most important organizational factors that influence over job satisfaction are job characteristics, which include job design, the tasks to be performed, the level of control employees have over their work, and opportunities for learning and development (Hackman, 1976). Working conditions, such as salary, benefits, work environment, job security, and work–life balance, are also crucial factors that can influence job satisfaction (Warr, 1987). Organizational culture plays a fundamental role; a positive organizational culture that values its employees, fosters communication and teamwork, and provides growth opportunities can contribute to higher job satisfaction (Schneider et al., 2013).

## 3. Hypotheses

Given the above, to achieve a comprehensive understanding of job satisfaction, this research includes factors from three levels of analysis; individual, group and organizational. Therefore, the following hypotheses are proposed:

**H1a.**  *Job demands defined as quantity will be inversely and significantly related to job satisfaction.*

**H2a.**  *Job demands defined as complexity will be inversely and significantly related to job satisfaction.*

**H1b.**  *Job resources defined as competency development will be directly and significantly related to job satisfaction.*

**H2b.**  *Job resources defined as the use of competencies will be directly and significantly related to job satisfaction.*

**H3b.**  *Job resources defined as decision-making latitude will be directly and significantly related to job satisfaction.*

**H1c.**  *Organizational identity will be directly and significantly related to job satisfaction.*

**H2c.**  *Group identity will be directly and significantly related to job satisfaction.*

## 4. Materials and Methods

### 4.1. Participants

The sample was obtained through a non-random, non-probability snowball sampling process. We directly contacted several organizations and invited them to share the link to our questionnaire with individuals who met the inclusion criteria. The inclusion criteria were: (a) being over 18 years of age, and (b) currently employed. No limitations were set regarding the type of employment or educational background for this study. The invitation included a brief explanation of the study’s purpose, the eligibility criteria, the voluntary nature of participation, and the right to withdraw at any time. Additionally, participants were informed that there was no economic compensation and that the study involved no foreseeable risks. No personal identifying data were collected. All participants accessed the survey anonymously and without any form of obligation or pressure. However, when analyzing the sociodemographic variables and correlations, we found that they did not significantly influence any of the proposed relationships. Therefore, we decided to omit these variables from the final model.

The sample consisted of 420 individuals (N = 420). Specifically, 49% of the sample were men (*n* = 214), while 51% were women (*n* = 216). Regarding age, the mean age of our sample was 41.58 years (SD = 9.95). Analyzing age by gender, we found that the mean age for men was 41 years (SD = 9.87), while similarly, the mean age for women was 42 years (SD = 10.02). Concerning educational level, our sample was primarily composed of individuals with a university degree (*n* = 202; 48.09%). The least frequent category was that of individuals holding a secondary education diploma (*n* = 34; 8.09%). Table 1 provides a more detailed distribution of our sample in relation to educational level.

Regarding the differences in educational level between men and women, we can observe that in both groups, the predominant academic level is that of a graduate (men, *n* = 91; 44.17%; women, *n* = 111; 51.87%), with a higher proportion of women holding a university degree or higher education.

Regarding the type of contract, our sample consisted predominantly of individuals with permanent contracts (*n* = 335; 79.76%), while the remaining participants had temporary contracts (*n* = 85; 20.24%). Table 2 presents the detailed distribution by gender.

It can be observed that, in relation to this variable, the distribution is quite similar between men and women.

Finally, the last sociodemographic variable of interest that we collected in our research referred to the presence of dependents of the worker. Specifically, to simplify the presentation, we categorized this variable into 0, 1, 2, and 3 or more dependents. Delving into this variable, the majority of our sample did not have dependents (*n* = 235; 55.95%), while the lowest frequency was for individuals who had 3 or more dependents (*n* = 40; 9.52%). Table 3 shows the distribution of this category by gender.

### 4.2. Instruments

For the rigorous evaluation of the variables included in the study, the following scales, translated and validated into Spanish, were used.

Job Content: The evaluation of job content was carried out using the Spanish adaptation (Agüir et al., 2001) of the Job Content Questionnaire (R. A. Karasek, 1985). This questionnaire measures three factors (demands, resources, and support), of which, following the model of Baker and Demerouti, two were selected (demands-resources). Here, it is important to argue that support is considered a resource… To gain a more comprehensive understanding of job demands and resources, the approaches of Bayona et al. (2015) were followed, who found that both demands and resources have multiple components. Specifically, we used, within job demands, the number of tasks (3 items) and task complexity (3 items). Regarding control, we used decision-making margin (3 items), the ability to utilize competencies (items 6, 9, 11), and the capacity for competency development (3 items). The original scale has shown that demands have a reliability of (0.74), control shows a reliability of (0.74), and support has shown a reliability of (0.88). In our sample, the demands factor showed a consistent reliability (0.80), while the control factor showed a good reliability as well (0.88). The selection of 3 items for each factor was made based on the significance of their content and the percentage of variance explained by the factor.

Job Satisfaction: To evaluate this variable, the Spanish validation (Fernández-Muñoz & Topa, 2018) of the Brief Scale of Affective Job Satisfaction (Thompson & Phua, 2012) was used. This brief scale, consisting of one factor and four items, helps assess the affective aspect of job satisfaction. Responses are rated on a 5-point Likert scale, with 1 = strongly disagree and 5 = strongly agree. To avoid acquiescence bias, the questionnaire includes three items that act as distractors and are not included in the final assessment. The reliability indices in the Spanish validation studies were above 0.80. In our sample, the reliability index yielded very good values, =0.87.

Social Identification, Group, and Organizational Identity: To assess this variable, an adapted version of the Organizational Identification Scale by Mael and Ashforth (1992) was used. Specifically, the recommendations of Topa and Morales were followed to adapt the scale for the purposes of this research. In the original studies, the Cronbach’s Alpha value was greater than 0.80. In our research, we found 0.88 for the factor of identification with the work group and 0.91 for the factor of identification with the organization. Responses were rated on a 7-point Likert scale (1 = strongly disagree; 7 = strongly agree).

### 4.3. Procedure

This study followed a cross-sectional survey design. Participants were recruited using a non-probability convenience and snowball sampling strategy. This research was approved by the National University of Distance Education (UNED) ethics committee pci-2023-8723F. First, individuals who were currently employed were contacted to invite them to participate in the study. The study was completed online through the Qualtrics application. The link to the questionnaire included the informed consent form, which provided the necessary information to ensure the ethical participation of the individuals in the research. Specifically, participants were informed about the research objectives, anonymity, confidentiality, and the voluntary nature of their participation. Additionally, participants were reminded of their right to withdraw from the study at any time and were provided with an email address for further information if desired.

### 4.4. Data Analysis

This research employs a cross-sectional survey design to conduct a MIMIC (Multiple Indicators Multiple Causes) analysis. Data analyses were conducted using JASP Software version 0.18. First, descriptive statistics, frequency tables, and correlations were calculated. After confirming the viability of a MIMIC model, the corresponding module was used for this process. To assess the model’s viability and fit, several indicators were used, as recommended in the scientific literature (Hu & Bentler, 1999). Specifically, the chi-square statistic was used for model testing. Additionally, the Comparative Fit Index (CFI), TLI, and GFI were applied, with values greater than 0.90 reflecting a good model fit (Hu & Bentler, 1999; Schermelleh-Engel et al., 2003). The Root Mean Square Error of Approximation (RMSEA) was considered, where values below 0.05 indicate adequate fit, and the Standardized Root Mean Square Residual (SRMR), with values below 0.08, indicates the suitability of the model.

## 5. Results

The MIMIC model was theorized taking into account the multifactorial nature of job satisfaction, as well as recent advancements in the field of job content. After reviewing the scientific literature, we identified three main variables that could help predict satisfaction. First, job content, not only in its dimensions of demands, control, and support, as justified by the classic research of R. A. Karasek (1979). Second, organizational identification and identification with the work group. Echoing advances proposed by authors such as Bakker and Demerouti, we have refined certain dimensions of job content (expand and revise). These variables have been related in the literature to job satisfaction, but the novelty of our work lies in understanding their contribution when placed in a model alongside organizational identity.

The first analysis performed was the correlation between the variables. The results can be seen in Table 4.

**Figure 1:**
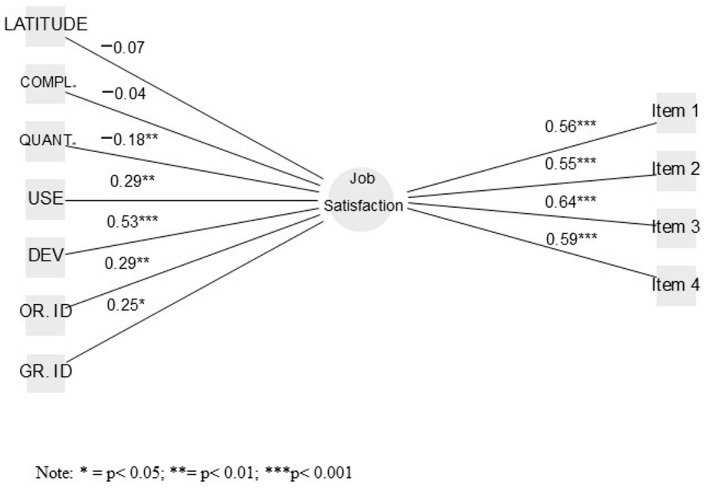
MIMIC model predicting Job Satisfaction. Note: OR.ID: Organizational Identification, GR.ID: Group Identification, DEV: development, QUANT.: Quantity, COMPL.: Complexity, USE: Competency use.

The theoretical model proposed for our study is shown in Figure 1. This model fitted the data in a statistically significant manner: χ^2^(234) = 1113.481, *p* < 0.001. CFI = 0.983, GFI = 0.997, TLI = 0.975, SRMR = 0.023, and RMSEA = 0.043. As we can see, all values are appropriate for estimating the model’s fit to the data. All predictors, except for task complexity and decision latitude, were statistically significant, with significance indices lower than *p* < 0.05. Moreover, all indicators were statistically significant with a *p*-value lower than *p* < 0.001, making them reliable indicators of the construct.

Based on this, we can affirm that we have sufficient evidence to support the following hypotheses: Job demands defined in terms of quantity (H1a) are inversely and statistically significantly related to job satisfaction. Job resources defined as both competence development (H1b) and the use of these competencies (H2b) are directly and statistically significantly related to job satisfaction. Lastly, both organizational identity (H1c) and group identity (H2c) are statistically significantly related to job satisfaction.

In contrast, we did not find sufficient evidence to accept the following hypotheses: Job demands defined in terms of complexity (H2a) are inversely and statistically significantly related to job satisfaction. Job resources defined in terms of decision latitude at the workplace (H3b) are directly and statistically significantly related to job satisfaction.

Regarding the strongest predictors of job satisfaction, the first is the ability to develop competencies (β = 0.525, *p* < 0.001). Next, organizational identity emerges as the following predictor in terms of explained variance (β = 0.285, *p* < 0.01). The ability to use competencies at the workplace ranks next in the percentage of explained variance for job satisfaction (β = 0.287, *p* < 0.01). Following this variable, but in a negative direction, we find physical demands (β = −0.175, *p* < 0.05). The last significant factor is group identity (β = 0.250, *p* < 0.05). In our model, neither task intensity (β = −0.038, *p* > 0.05) nor decision latitude (β = −0.072, *p* > 0.05) were significant. The total explained variance by the overall model is quite large (R^2^ = 0.442).

## 6. Discussion

The main objective of this study was to examine how job characteristics—specifically job demands and job resources—along with organizational and group identification, predict affective job satisfaction. By integrating elements from the Job Demands–Resources model and social identity theory, we aimed to offer a comprehensive explanation of the factors influencing employees’ satisfaction at work.

The results of this study partially confirm the proposed hypotheses, showing that job demands and resources, as well as organizational and group identification, are significantly related to job satisfaction. As proposed in the hypotheses, job demands (quantity and complexity) showed an inverse relationship with job satisfaction, while job resources (competency development, competency use, and decision latitude) showed a positive relationship. Additionally, both organizational and group identification positively contributed to job satisfaction.

Our research aligns with other previous studies (Fernández-Salinero et al., 2019, 2020), where we demonstrated that employees with a high degree of organizational and group identification experience greater satisfaction. This suggests that organizational identification is not only a resource in itself but also a protective factor against burnout and other negative effects of job stress as previous research show (Quesada-Puga et al., 2024).

Although decision latitude and task complexity were theoretically considered important predictors of job satisfaction, our results did not support their statistical significance. One possible explanation is that the influence of these variables may depend on specific contextual factors, such as organizational culture or job type. Future research should analyze these variables to gain a deeper understanding of these factors in liberal professions or in public administration. Linked to this, it is also possible that, in our sample, decision-making autonomy was not perceived as particularly impactful when compared to more tangible resources. Maybe future research should consider leadership styles and evaluating its differential impact. Regarding task complexity, some studies suggest that complexity may not have a linear effect on satisfaction; while moderate levels can be motivating, excessive complexity might lead to frustration, especially in environments lacking adequate support (Xanthopoulou et al., 2007). Future research should explore whether these variables play a more nuanced or indirect role, potentially moderated or mediated by other job or personal factors.

This study contributes to the theoretical literature on job satisfaction by integrating the Job Demands–Resources model with social identity theory in a single explanatory model. Our findings highlight the importance of distinguishing between different dimensions of job demands (quantity vs. complexity) and resources (competency development vs. use), providing a more refined understanding of how these elements interact to shape affective job satisfaction. Moreover, the results support the relevance of both organizational and group identification as distinct but complementary psychosocial resources, emphasizing the need to consider social identity variables in models of workplace well-being.

However, it is important to note that the JD-R model has been criticized for its limited contextual integration and its predominantly individual-level focus (Schaufeli & Taris, 2013). The model tends to treat job demands and resources as isolated perceptions rather than as elements embedded in broader organizational or social environments. Work behavior is embedded within broader organizational and social structures, and it is increasingly difficult to fully understand these phenomena by relying solely on individual perceptions. While our study adopts an individual-level approach, this is a limitation of our approach. Future research should adopt a broader and more comprehensive perspective that accounts for the influence of contextual, structural, and collective factors on job satisfaction. Future researchers may find a broader optics in terms such as worker collective or operational leeway which have been considered as crucial for preserving collective health and performance. Managers should consider not only physical and psychological demands and resources but also the social dynamics within the organization. Fostering an environment where employees identify with both their workgroup and the organization could significantly improve job satisfaction, reducing absenteeism and turnover, as evidenced in the literature (Van Dick et al., 2004). This should also be considered in remote environments, where organizational and group identification is cultivated differently. Research like that of Wang et al. (2021) shows how remote workers remain in their jobs due to perceived benefits rather than emotional bonds with colleagues or the organization. Additionally, the job demands–resources theory continues to provide important scientific evidence suggesting that when there is a match between job demands and resources, employees experience lower levels of stress, as well as greater psychological well-being and job satisfaction (Dishon-Berkovits et al., 2024). In contrast to competency utilization, our study aligns with findings from other studies indicating that when employees cannot use their competencies, the risk of burnout significantly increases, and job satisfaction decreases (Lee & Jo, 2023; Huang et al., 2016).

Our research is also aligned with current studies showing that opportunities for competency development and effective application have a significant impact on job satisfaction (Jaimes et al., 2024). In our case, these two factors have proven crucial in predicting job satisfaction.

From a practical standpoint, the results suggest that organizations should prioritize the development and effective use of employee competencies to enhance job satisfaction. Providing opportunities for skill development and ensuring that employees can apply their competencies in meaningful ways may reduce the negative impact of quantitative job demands. In addition, promoting organizational and group identification through inclusive leadership, transparent communication, and shared goals may improve employees’ emotional connection with their work and their workplace. These interventions could be especially beneficial in remote or hybrid work environments, where identification processes may be weaker or more fragmented.

Future research should pay more attention to the specificities of organizational and group identification for people who telework or are in hyper-connected environments, where the competencies to develop and practice may differ from in-person environments.

Our study also presents certain limitations. First, the cross-sectional design of the study prevents establishing clear causal relationships between variables. Future research could benefit from longitudinal designs that allow evaluating how these relationships evolve over time. Second, the sampling procedure, based on non-probability convenience and snowball techniques, limits the generalizability of the findings. As a result of this methodology, it was not possible to determine the exact number of invitations sent or the response rate, which restricts our ability to assess potential sampling biases. Third, the online data collection method, while efficient, may have excluded individuals without reliable internet access or those less inclined to participate in digital surveys (e.g., older workers or certain socioeconomic groups). This self-selection bias could limit the generalizability of our findings. Future studies could combine online methods with targeted offline recruitment to improve representativeness. Additionally, we did not include contextual variables such as company size or working modality. Future research should consider incorporating these contextual elements to better understand how working conditions may interact with job demands, resources, and identification processes. It would be interesting to replicate this study in different organizational and cultural contexts to validate these findings. Finally, precisely because we do not know the percentage of the sample working remotely, we could not establish groups to compare whether there are statistically significant differences in the variables of interest and their relationships.

## 7. Conclusions

Our model offers a comprehensive and global view of the influence of job demands, job resources, and organizational and group identification on job satisfaction. The complexity of tasks (demand) and decision latitude (control resource) did not show a statistically significant influence on job satisfaction. This is crucial, as it may allow managers to develop more tailored and healthier job positions.

## Figures and Tables

**Table 1:** Frequencies of Educational Level.

Educational Level	Frequency	Men	Women
Primary	36	25	11
Secondary	34	17	17
Vocational training	81	49	32
University degree	202	91	111
Postgraduate	67	24	43
Total	420	206	214

**Table 2:** Frequencies of Type of Contract.

Type of Contract	Frequency	Men	Women
Permanent	335	161	174
Temporary	85	45	40
Total	420	206	214

**Table 3:** Frequencies of People in Charge.

People in Charge	Frequency	Men	Women
0	235	101	134
1	60	34	26
2	85	47	38
3 or more	40	24	16
Total	420	206	214

**Table 4:** Correlation matrix between variables.

Variable	J.S.	OR. ID	GR. ID	QUANT.	COMPL.	LATITUDE	USE
1. J.S.	—						
2. OR. ID.	0.468 ***	—					
3. GR. ID.	0.449 ***	0.806 ***	—				
4. QUANT.	−0.035	−0.064	−0.074	—			
5. COMPL.	0.092	−0.037	−0.018	0.686 ***	—		
6. LATITUDE	0.332 ***	0.213 ***	0.174 ***	0.066	0.291 ***	—	
7. USE	0.434 ***	0.246 ***	0.239 ***	0.192 ***	0.361 ***	0.644 ***	—
8. DEVELOPMENT	0.503 ***	0.325 ***	0.310 ***	0.152 **	0.369 ***	0.660 ***	0.707 ***

Note: J.S.: Job Satisfaction, OR.ID: Organizational Identification, GR.ID: Group Identification, QUANT.: Quantity, COMPL.: Complexity, USE: Competency use. ** = *p* < 0.01 *** = *p* < 0.001.

## Data Availability

The database would be made available upon a reasonable request.
